# Somatic genome doubling determines fertility in maize haploids of inbred A427

**DOI:** 10.3389/fpls.2026.1937834

**Published:** 2026-09-21

**Authors:** Recep Yavuz, Somak Dutta, Memiş Bilgici, Ursula K. Frei, Siddique I. Aboobucker, Thomas Lubberstedt

**Affiliations:** 1 Department of Agronomy, Iowa State University of Science and Technology, Ames, IA, United States; 2 Department of Statistics, Iowa State University of Science and Technology, Ames, IA, United States; 3 Department of Plant and Soil Sciences, University of Kentucky, Lexington, KY, United States

**Keywords:** flow cytometry, haploid male fertility, maize, spontaneous genome doubling, stomatal length

## Abstract

Doubled haploid (DH) technology underpins modern maize breeding, yet widespread male sterility in haploids necessitates toxic chemical chromosome doubling. Spontaneous haploid genome doubling (SHGD) offers a biological alternative, but its developmental dynamics and cellular basis remain unresolved. Here, using the high haploid male fertility (HMF) genotype A427, we integrate flow cytometry, stomatal phenotyping and whole-plant performance analyses across three independent experimental years involving 2,924 individual measurements to characterize the temporal progression of genome doubling. Fertile and sterile A427 haploids showed progressively divergent nuclear DNA-content profiles during vegetative development, with fertile plants exhibiting increasing enrichment of 2C and 4C nuclear fractions at later leaf stages. Stomatal length at the 5th leaf stage (L5) strongly discriminated between plants subsequently classified as male fertile and male sterile (apparent AUC = 0.886), providing a proof-of-concept for early, non-destructive selection. Diploidization at the 15th leaf stage (L15) correlated with vegetative vigor and reproductive architecture, linking cellular ploidy dynamics to whole-plant performance. These findings support a developmental association between somatic diploidization and male fertility in A427 and provide a framework for evaluating chemical-free selection strategies, while validation across independent populations, genetic backgrounds and environments will be required before broader breeding application.

## Introduction

Doubled haploid (DH) technology has accelerated genetic gain in maize breeding by enabling rapid production of completely homozygous lines. However, most maize haploids exhibit poor vigor and male sterility, necessitating haploid genome doubling by using toxic chemicals such as colchicine. Spontaneous haploid genome doubling (SHGD) represents a promising alternative that allows DH line production without chemical intervention (Kleiber et al., 2012; Wu et al., 2014; Ren et al., 2020). Despite its potential, the developmental timing and cellular basis of SHGD remain poorly understood.

Classical studies on ploidy effects in plants have shown that haploids display reduced vigor and fertility (Chase, 1964; Guo et al., 1996; Miller et al., 2012), a phenomenon later conceptualized as *haploid frailty* (Yavuz et al., 2025; Fakude et al., 2025). In maize, more than 90% of haploid genotypes display extremely low haploid male fertility (HMF) (Kleiber et al., 2012; Sugihara et al., 2013). Nevertheless, substantial genetic variation for HMF exists, and multiple inbred lines with elevated levels of haploid fertility have been identified, demonstrating that HMF is a selectable trait (Boerman et al., 2020; De la Fuente et al., 2020; Foster et al., 2024).

Importantly, SHGD exhibits incomplete penetrance: even within genetically uniform haploid lines, genome doubling occurs only in a subset of individuals or cell lineages. Consequently, plants differ in the extent of somatic diploidization, generating a continuum from complete sterility to full fertility. This probabilistic, developmentally dynamic behavior explains how sterile and fertile haploids can arise from the same genetic background.

To date, no genes controlling HMF have been identified in maize. In contrast, studies in *Arabidopsis thaliana* have uncovered genes that confer haploid fertility through abnormal meiotic processes, leading to unreduced gametes and fertile pollen (Aboobucker et al., 2023). In maize haploid plants derived from inbred line A427, phenotypic and cytological evidence suggest that haploid genome doubling is promoted by mitotic processes occurring throughout plant development, resulting in chimeric haploids with enhanced vigor and fertility (Yavuz et al., 2025).

This study aimed (i) to determine the developmental timing and cellular context of spontaneous haploid genome doubling in haploids derived from maize inbred A427, (ii) to quantify how early somatic genome doubling influences cellular ploidy dynamics, haploid vigor, and male fertility, and (iii), to evaluate whether mitotic genome doubling generates chimeric haploids with progressively increasing diploid cell fractions. This work seeks to enable early identification of likely fertile haploid plants, contributing to more efficient and sustainable DH breeding systems without the need for chemical haploid genome doubling.

## Materials and methods

### Plant materials

The genotypes used in this study were haploid A427 (n), diploid A427 (2n), diploid Wf9 (2n), haploid Wf9 (n; sterile), and diploid W22 (2n; control). Diploid A427 (2n) and Wf9 (2n) seeds used as parental and control material were obtained from the USDA-ARS North Central Regional Plant Introduction Station (NCRPIS, Ames, IA, USA), as described previously (De la Fuente et al., 2020). Haploid seeds were generated by crossing these inbred lines as female parents with the haploid inducer line BHI306. Putative haploids were identified manually at the seed stage based on R1-Navajo anthocyanin pigmentation. R1-Navajo-selected seeds were not independently confirmed as haploids by flow cytometry or chromosome counting before inclusion; rather, nuclear DNA content was subsequently characterized by flow cytometry at multiple vegetative stages as part of the study. Seeds of W22 were obtained from the Maize Genetics Cooperation Stock Center (Urbana, IL, USA) and were used as the diploid internal standard for flow cytometry.

A427 (n) is characterized by high HMF and a strong capacity for SHGD (De la Fuente et al., 2020), whereas Wf9 (n) exhibits near-complete haploid male sterility. Therefore, these genotypes were selected to represent contrasting haploid male fertility phenotypes.

Plants were grown in pots under fully controlled greenhouse conditions at the Iowa State University Department of Horticulture (Ames, IA, USA) from 2023 to 2025. Environmental conditions were maintained at 25 ± 2 °C during the day and 20 ± 2 °C at night, with a relative humidity of 60-70% and a 16 h light/8 h dark photoperiod. Plants were irrigated and fertilized regularly to avoid water or nutrient stress.

### Experimental design

Greenhouse experiments were conducted over three consecutive years, each representing an independent experimental run. Haploid genotypes A427 (n) and Wf9 (n), together with diploid controls A427 (2n), Wf9 (2n) and W22 (2n), were grown in pots under controlled conditions and arranged in a completely randomized design. Each year, individual plants were treated as experimental units. Male fertility status was determined at reproductive maturity during the pollen-shedding period. Plants displaying developed tassels with observable pollen production/shedding were classified as male fertile, whereas plants that failed to produce or shed detectable pollen were classified as male sterile (**Supplementary Figures 11**, **12**). Pollen viability staining (e.g., Alexander or I2-KI staining) was not performed; therefore, fertility classification represents phenotypic male fertility/pollen shedding rather than a quantitative assay of pollen viability. Fertility status was not assigned during vegetative development. Measurements collected at L1, L5, L10 and L15 were grouped retrospectively according to the fertility phenotype subsequently observed at maturity.

Leaf samples were collected at the L1, L5, L10 and L15 leaf stages for flow cytometric quantification of 1C, 2C and 4C nuclear fractions. Stomatal density (SD) and stomatal length (SL) were measured at L5, L10 and L15. Agronomic traits recorded at maturity included plant height (PH), ear height (EH), spike length (SpL), spike diameter (SpD), flag leaf length (FLL), flag leaf width (FLW), tassel length (TL), and tassel branch number (TB). The 4C:1C ratio was calculated per individual plant at L15.

Each year, 36 plants were established. Plants that did not survive to the pollen stage were excluded from the final dataset. After quality filtering, 23 plants from year 1, 23 plants from year 2 and 24 plants from year 3 were retained, resulting in 70 plants and a total of 2,924 observations included in the analyses. The final set of 70 plants comprised 45 A427 (n), 16 Wf9 (n), 4 A427 (2n), 3 Wf9 (2n), and 2 W22 (2n) plants. Agronomic traits and stomatal traits expect for plant height were recorded in years 2 and 3 only. Degrees of freedom (DF) varied among traits due to year-specific measurements and missing observations after quality filtering.

### Flow cytometry

Nuclear DNA content was quantified using flow cytometry following the protocol described by Doležel et al. (2007). Approximately 20–30 mg of fresh leaf tissue was excised and finely chopped with a razor blade in ice-cold Galbraith’s buffer (Galbraith et al., 1983). The resulting nuclear suspension was filtered through a 42 µm nylon mesh and stained with propidium iodide (50 µg mL^−1^) in the presence of RNase (50 µg mL^−1^).

Sample preparation was performed in the laboratories of the Iowa State University Department of Agronomy, and flow cytometric analyses were conducted at the Iowa State University Flow Cytometry Facility of the Office of Biotechnology (https://www.biotech.iastate.edu/). For each sample, at least 5,000 nuclei were analyzed. Peaks corresponding to 1C, 2C, and 4C nuclear DNA content were identified after nuclear-quality gating. Approximately 15% of recorded events fell outside the clearly defined peak regions or could not be unambiguously assigned to one of the 1C, 2C, or 4C DNA-content classes and were therefore excluded from subsequent analyses. This filtering criterion was applied consistently across samples to remove ambiguous/background events rather than to selectively exclude any specific ploidy class.

### Stomatal phenotyping by epidermal imprint analysis

SD and SL were quantified from epidermal impressions taken from the abaxial surface of the L5, L10, and L15 leaves using the clear nail polish imprint technique (Weyers and Johansen, 1985; Wu and Zhao, 2017). A thin layer of transparent nail varnish was applied to the central region of the leaf blade, allowed to dry (10 min), and carefully peeled off using clear adhesive tape. The imprints were examined under a light microscope at 400x magnification. For each leaf, at least three non-overlapping microscopic fields were imaged from the mid-lamina region. All clearly visible stomata within each selected microscopic field were measured; no fixed subsample of stomata was selected. Stomatal density was calculated as the number of stomata per unit leaf area (mm²), and stomatal length was determined by measuring guard cell length from digital micrographs using image analysis software.

### Statistical analysis

Differences between fertile and sterile haploid plants were evaluated using analysis of variance (ANOVA), with experiment included as a fixed effect in the model. Specifically, for each trait, the following linear model was used:


$$Y_{ijk}=\mu +b_{i}+f_{j}+\epsilon_{ijk},$$

where *Y_ijk_* is the trait value of the *k*th replicate of the *j*th group (fertile or sterile) in the *i*th year; *μ* is the overall mean; *b_i_* represents the year effects; *f_j_* represents the fertility effects; and *ϵ_ijk_* are independent $N\left(0,\sigma^{2}\right)$ random errors. Subsamples from the same leaf were averaged prior to model fitting. Estimated marginal means were obtained from the fitted model and used to estimate and test contrasts between fertile and sterile groups.

Pearson correlation coefficients were calculated to assess associations between ploidy- related variables and agronomic traits after adjusting for year effects. Statistical significance was evaluated using the cor.test function in R, and multiple p-values were adjusted for false discovery rate using the Benjamini–Hochberg method.

To assess the predictive performance of individual traits for fertility status, receiver operating characteristic (ROC) analyses were performed, and the area under the ROC curve (AUC) was calculated for each trait (**Table 1**). When multiple predictors were combined, fertility status was modeled using binomial logistic regression and model performance was evaluated using ROC analysis and corresponding AUC values. Because the ROC models were developed and evaluated using the same experimental dataset, these AUC values represent apparent/internal discriminatory performance rather than independently validated predictive accuracy. No independent validation population or cross-validation procedure was used; therefore, the reported AUC values should be interpreted as exploratory proof-of-concept estimates requiring validation in independent populations, genotypes, and environments.

**Table 1:** Estimates of mean trait differences (fertile minus sterile), standard errors (s.e.), degrees of freedom (DF) and FDR-adjusted P values from ANOVA models including experimental year as a fixed effect.

Trait	Estimate	SE	DF	p-value (adj.)	AUC
L1.1C (%)	-2.99	3.33	59	0.39	0.478
L1.2C (%)	-3.62	1.99	59	0.096	0.673
L1.4C (%)	5.93	2.32	59	0.029	0.667
L5.1C (%)	-4.48	3.73	66	0.26	0.601
L5.2C (%)	-2.41	2.21	66	0.30	0.591
L5.4C (%)	4.99	2.63	66	0.089	0.618
L10.1C (%)	-9.67	3.61	65	0.022	0.661
L10.2C (%)	4.21	2.29	65	0.096	0.649
L10.4C (%)	5.40	1.96	65	0.02	0.651
L15.1C (%)	-13.77	3.21	65	< 0.001	0.748
L15.2C (%)	8.06	2.21	65	0.0028	0.768
L15.4C (%)	4.45	1.25	65	0.003	0.742
L5.Stomatal density (SD)	-1.90	0.86	44	0.054	0.699
L5.Stomatal length (SL)	32.67	6.78	44	< 0.001	0.886
L10.SD	-1.72	1.10	44	0.15	0.652
L10.SL	18.68	8.51	44	0.054	0.665
L15.SD	-2.06	1.05	44	0.086	0.661
L15.SL	22.68	7.41	44	0.012	0.757
Tassel branch (TB)-cm	-1.86	1.42	44	0.235	0.465
Tassel length (TL)-cm	10.07	1.84	44	< 0.001	0.880
Plant height (PH)-cm	33.79	7.02	66	< 0.001	0.680
Ear height (EH)-cm	-0.66	4.65	44	0.89	0.574
Flag leaf length (FLL)-cm	9.40	4.10	44	0.051	0.683
Flag leaf width (FLW)-cm	1.43	0.48	44	0.014	0.743
Spike Length (SpL)-cm	17.32	5.41	44	0.0095	0.725
Spike diameter (SpD)-cm	0.25	0.11	44	0.051	0.692

Results are presented for all plants included in the experiment. AUC values are reported on a 0–1 scale

All statistical analyses were conducted in R Statistical Software (R Core Team, 2025). Estimated marginal means were obtained using the emmeans package (Lenth, 2025), ROC analyses were performed using the pROC package (Robin et al., 2011), correlations were tested using the cor.test function, and p-values were adjusted using the p.adjust function.

## Results

### Developmental divergence in somatic genome doubling between fertile and sterile haploids

Flow cytometric analysis of L15 tissue from A427 haploid plants revealed contrasting nuclear DNA profiles between fertile and sterile individuals. In fertile haploids, sequential gating of intact nuclei yielded a high-purity population (>90%), and the DNA histogram showed a substantial 2C (diploid DNA content) fraction (27.0%) together with detectable 4C (replicated diploid DNA content) nuclei (8.15%), alongside a reduced 1C peak (29.3%). This pattern indicates active somatic genome doubling and a chimeric ploidy structure at L15 (**Figure 1**). In contrast, the sterile haploids displayed a predominantly haploid nuclear profile, characterized by a dominant 1C (haploid DNA content) peak (49.3%) and markedly reduced 2C (22.8%) and 4C (3.84%) fractions. The limited accumulation of diploidized nuclei is consistent with restricted somatic genome doubling in sterile plants (**Figure 2**). Together, these representative profiles illustrate the developmental divergence in ploidy composition at L15 and support the quantitative differences reported between fertile and sterile haploids.

**Figure 1:**
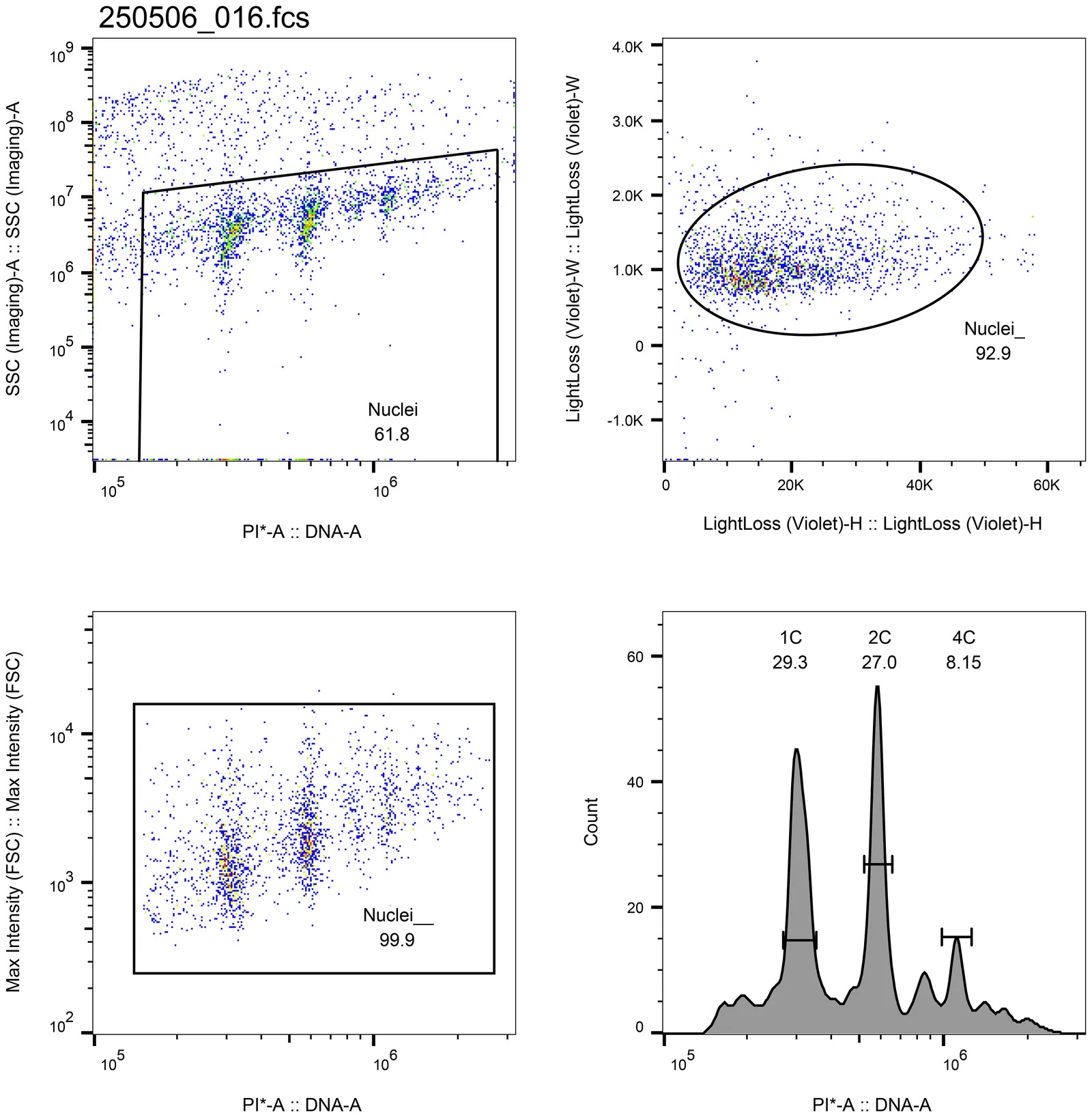
Flow cytometric profiles of L15 leaf nuclei from A427 haploid plants (fertile).

**Figure 2:**
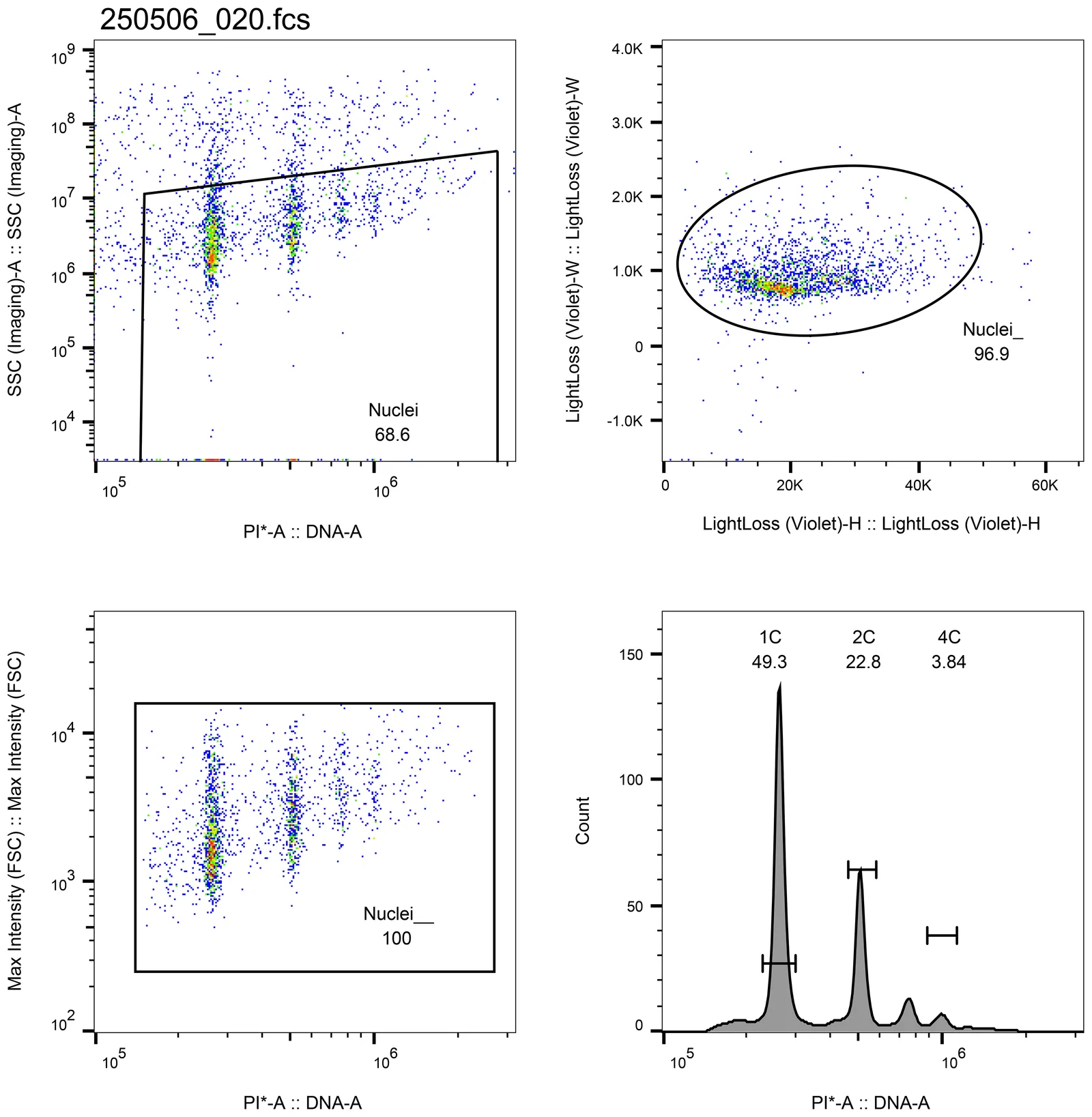
Flow cytometric profiles of L15 leaf nuclei from A427 haploid plants (sterile).

The x-axis represents relative fluorescence intensity (DNA content), while the y-axis indicates the number of nuclei (counts) in both **Figures 1** and **2**.

Sterile haploids exhibited significantly higher proportions of 1C nuclei than fertile haploids at the 10th leaf stage (L10) (46.8% versus 37.1%; adjusted P = 0.022), and this divergence became more pronounced at L15 (50.4% versus 36.7%; adjusted P = 3.9 × 10^−4^). Correspondingly, the proportion of 2C nuclei was significantly higher in fertile haploids at L15 (33.9% versus 25.9%; adjusted P = 0.0028), and the 4C fraction also differed at L15 (adjusted P = 0.0030).

By contrast, earlier developmental stages -1st leaf stage (L1) and L5- showed no statistically significant differences in 1C or 2C fractions after false discovery rate (FDR) correction. Together, these results demonstrate a developmental shift in fertile haploids characterized by a progressive reduction in 1C nuclei accompanied by increases in 2C and 4C fractions, whereas sterile haploids maintained predominantly haploid nuclear profiles throughout development. Effect sizes were substantial at L15, with the estimated difference in 1C fraction (fertile minus sterile) reaching -13.77 percentage points ± 3.21 (mean ± s.e.), indicating a marked enrichment of diploidized nuclei in fertile plants at later developmental stages (**Figure 3**; **Supplementary Figures 1**–**8**).

**Figure 3:**
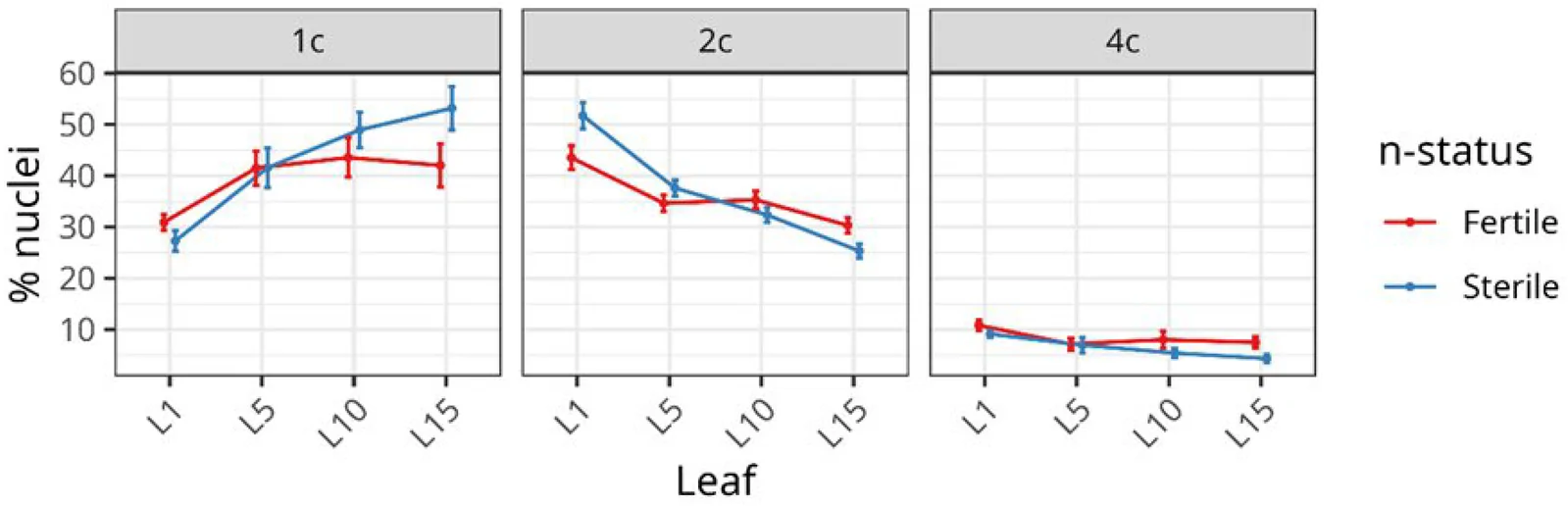
Comparison of fertile and sterile n genotypes (mean ± SE) at ploidy levels 1C, 2C, and 4C across leaf stages (L1, L5, L10, L15).

### Predictive performance of ploidy levels and morphological traits

To assess whether developmental traits were associated with eventual fertility status (male fertile versus sterile), we evaluated the discriminatory performance of nuclear DNA fractions across successive leaf stages using receiver operating characteristic analyses. These analyses were exploratory and retrospective because fertility groups were assigned only after reproductive maturity. In this context, L15 ploidy profiles were evaluated primarily as evidence of developmental divergence before reproductive maturity, whereas L5 stomatal traits were evaluated as the earlier candidate markers with greater practical relevance for selection.

DNA content measurements from L1-L10, including derived ratios, showed limited apparent discriminatory performance for eventual fertility status (AUC range 0.47-0.65). In contrast, L15 ploidy fractions displayed improved discrimination (apparent AUC = 0.74-0.77), supporting developmental divergence in nuclear DNA-content profiles before reproductive maturity rather than defining L15 as an optimal early selection stage. Stomatal length measured at L5 exhibited strong apparent classification performance (AUC = 0.886), outperforming all cellular ploidy metrics in this dataset. Incorporation of the L15 1C fraction into the stomatal length model yielded only a marginal improvement (combined apparent AUC = 0.896), suggesting limited additive discriminatory information from L15 ploidy once L5 stomatal length was considered. These estimates were obtained from the same dataset used to develop the models and therefore require independent validation.

At the L5 stage, stomatal length (SL) differed markedly between fertile and sterile haploid plants. Male-fertile A427 (n) plants exhibited a mean SL of 131.7 µm, whereas male-sterile A427 (n) plants showed a mean SL of 92.1 µm (**Figures 4**, **5**). In controls, diploid W22 displayed the highest mean SL (163.8 µm), while haploid Wf9 showed the lowest SL (79.7 µm) (**Supplementary Figures 9**, **10**). Fertile A427 haploids therefore exhibited substantially longer stomata than sterile A427 haploids, with mean values shifted in the direction of the diploid control; however, statistical equivalence between fertile haploids and diploid controls was not tested. Thus, the stomatal data support an association between increased stomatal length, eventual male fertility, and elevated genome dosage without implying formal equivalence to the diploid phenotype.

**Figure 4:**
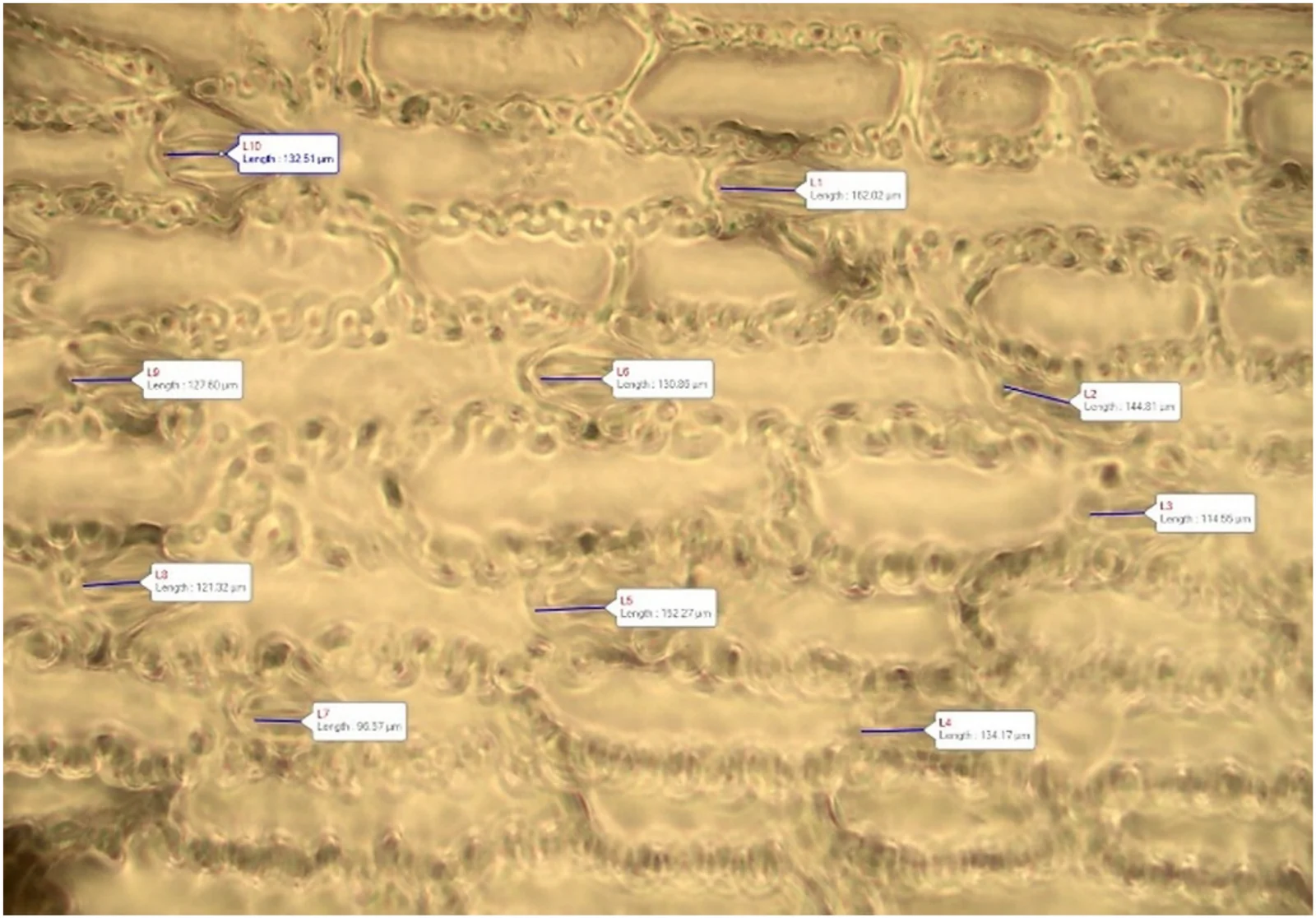
Stomatal length (SL) in male-fertile A427 (n) plants at L5 (mean = 131.67 µm).

**Figure 5:**
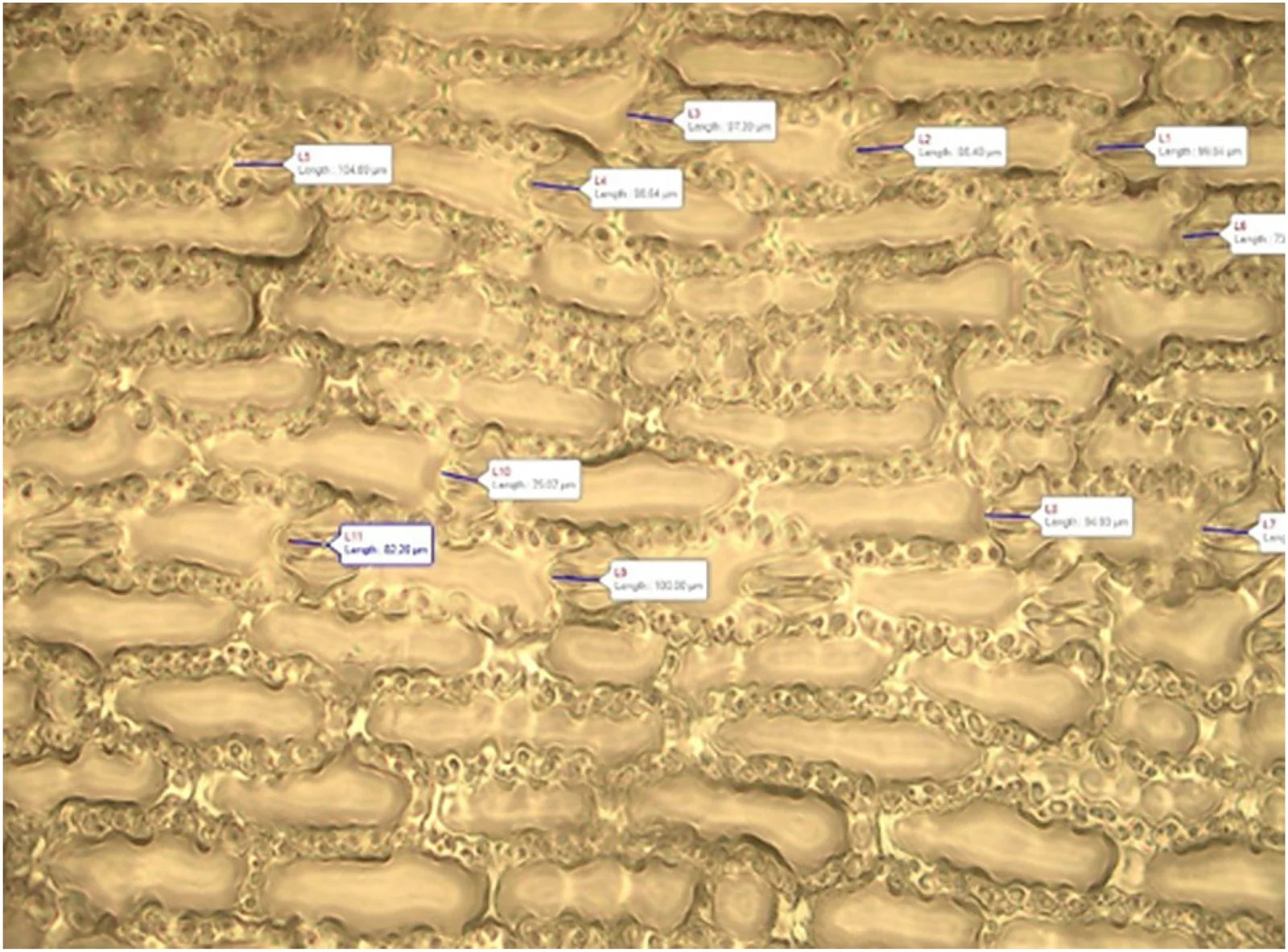
Stomatal length (SL) in male-sterile A427 (n) plants at L5 (mean = 92.1 µm).

At the optimal threshold determined by the Youden index within this dataset, stomatal length alone would eliminate approximately 50% of sterile plants while retaining >94% of fertile individuals (**Table 1**; **Figure 6**). This result demonstrates promising proof-of-concept discriminatory utility for early-stage selection in A427; however, because the threshold and performance were estimated retrospectively from the same dataset, independent validation is required before operational breeding use.

**Figure 6:**
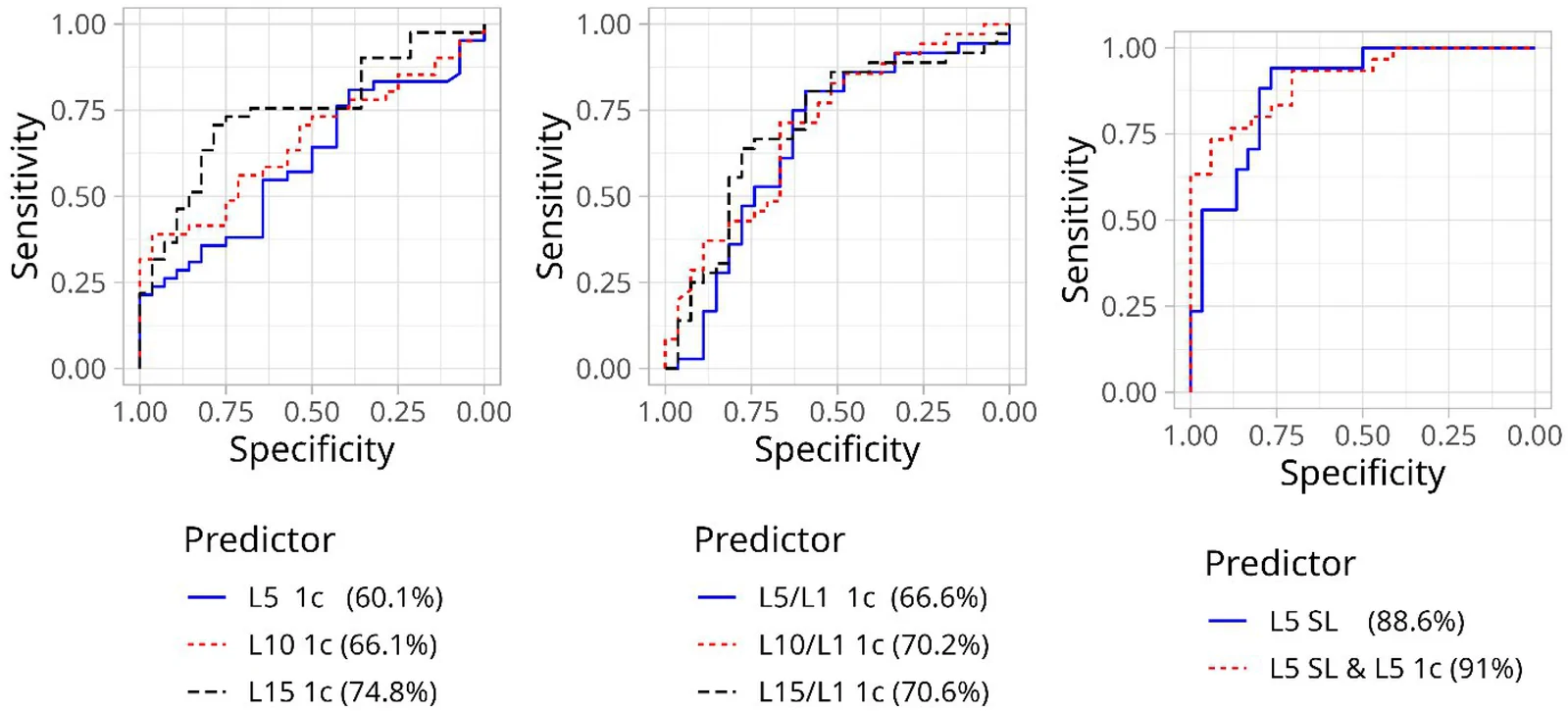
Receiver operating characteristic (ROC) curves for classification of fertility status. Left panel uses the 1C contents, the center panel uses the fold change in 1C contents from the first leaves, and the right panel uses the stomatal length (SL) of L5 with or without 1c contents from the L5. AUC values are shown in parentheses. Because model development and evaluation used the same dataset, these AUC values represent apparent/internal discriminatory performance.

### Association between ploidy and agronomic performance

Higher 4C:1C ratios at L15 were positively associated with several vegetative and reproductive traits. The strongest associations were observed for plant height (r = 0.57, adjusted P< 1 × 10^−4^) and tassel length (r = 0.58, adjusted P< 1 × 10^−4^), indicating moderate-to-strong positive correlations between diploid cell enrichment and vegetative growth. Significant positive correlations were also detected for ear height (r = 0.54, adjusted P = 2 × 10^−4^), flag leaf length (r = 0.53, adjusted P = 3 × 10^−4^), flag leaf width (r = 0.44, adjusted P = 0.0026), spike length (r = 0.44, adjusted P = 0.0026) and spike diameter (r = 0.53, adjusted P = 3 × 10^−4^). Tassel branch number showed only a marginal association (r = 0.29, adjusted P = 0.0570).

These results indicate that increased proportions of diploidized nuclei at L15 are broadly associated with enhanced vegetative growth and reproductive architecture (**Table 2**; **Figure 7**).

**Table 2:** Correlations between agronomic traits and 4C to 1C ratio at L15, along with FDR adjusted p-values for testing if the correlations are zero.

Trait	Cor. coef	p-value (adj)
TB	0.285	0.0570
TL	0.579	<0.0001
PH	0.565	<0.0001
EH	0.543	0.0002
FLL	0.526	0.0003
FLW	0.438	0.0026
SpL	0.444	0.0026
SpD	0.533	0.0003

Results are presented for all plants included in the experiment.

**Figure 7:**
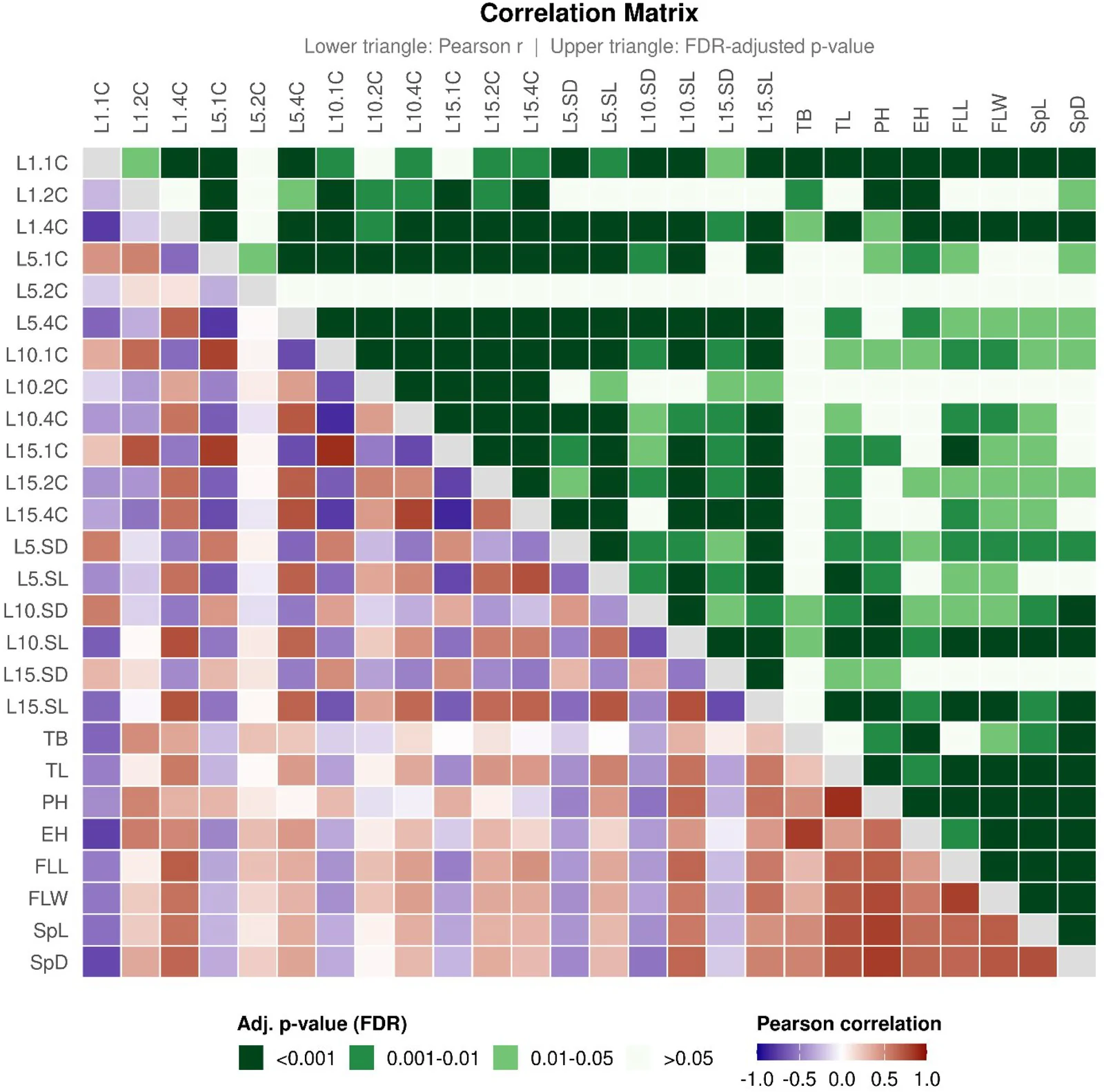
Correlation matrix showing relationships among nuclear DNA content and agronomic traits (PH, EH, TL, TB, FLL, FLW, SpL, SpD) in all plants included in the experiment. Pearson correlation coefficients are displayed in the lower triangle, while the upper triangle shows the corresponding significance levels (P values).

### Stable ploidy in diploid controls and persistent haploidy in sterile genotypes

Diploid control genotypes (A427, Wf9 and W22) exhibited stable 2C and 4C nuclear DNA distributions across all sampled leaf stages, with no detectables 1C enrichment. In contrast, the uniformly sterile haploid genotype Wf9 displayed persistent 1C nuclear peaks throughout development, with no detectable shift toward 2C or 4C fractions. This pattern supports a strong association between somatic genome doubling and male fertility status in haploid maize.

### Predictive power of cellular and morphological traits for fertility

Combining stomatal length with L15 leaf ploidy information only marginally improved apparent discriminatory performance. Within the A427 dataset, this suggests that stomatal length measured at the L5 leaf stage may provide an earlier and simpler candidate trait for distinguishing plants that subsequently become fertile or sterile. However, this finding is retrospective and has not been validated in an independent population. Correlation analyses further revealed that the 4C:1C ratio at the L15 leaf stage was strongly and positively associated with plant height, tassel length, flag leaf dimensions, and ear/spike-related traits (**Supplementary Figures 9**, **10**).

## Discussion

SHGD represents a central yet mechanistically heterogeneous step in DH technology in maize. Rather than arising from a single causal pathway, increasing evidence suggests that multiple independent biological routes can lead to spontaneous haploid genome doubling. In this study, we focused specifically on HMF, as male sterility constitutes the principal biological limitation in maize DH production. Haploid female fertility (HFF) is generally less limiting in maize breeding systems. Nevertheless, significant genetic variation for HFF exists and contributes as one of two major components of successful SHGD (Fakude et al., 2025). Thus, SHGD should be viewed as a composite outcome integrating both haploid male and female reproductive competence.

For HMF in particular, at least two conceptually distinct mechanisms are likely to operate. One route involves meiotic restitution events that generates unreduced gametes. Cytological analyses in maize have documented multiple meiotic pathways leading to diploid pollen formation (Zhang et al., 2022), and genetic analyses indicate that complex networks affecting chromosome segregation and spindle organization contribute to spontaneous doubling (Boerman et al., 2020). In *Arabidopsis thaliana*, specific genes such as *ps1* and *jas2* have been shown to promote haploid fertility via meiotic abnormalities that restore gamete functionality (Aboobucker et al., 2023), demonstrating that meiotic HMF can arise from defined molecular lesions.

A second, mechanistically distinct route may involve mitotic genome doubling occurring during vegetative development. Our data are consistent with a contribution of this somatic/mitotic pathway in the high-HMF genotype A427. Fertile haploids showed progressive reductions in 1C nuclei and corresponding increases in 2C and 4C fractions that become pronounced by the fifteenth leaf stage, whereas sterile haploids maintained persistently elevated 1C fractions across developmental stages. This divergence precedes reproductive maturation and therefore supports the hypothesis that somatic ploidy dynamics are established before reproductive development. However, flow cytometry of vegetative tissues does not directly visualize individual mitotic genome-doubling events, and direct cytological observations of pollen mother-cell meiosis were not performed. Consequently, the present study cannot quantitatively separate the contributions of somatic/mitotic doubling and meiotic restitution to male fertility.

Beyond fertility itself, the strong positive correlations between the L15 4C:1C ratio and multiple agronomic traits demonstrate that genome doubling scales with whole-plant vigor. Similar to observations that HMF and spontaneous doubling rate are heritable and genetically controlled traits (Molenaar et al., 2019), our results indicate that the extent of somatic diploidization quantitatively influences vegetative architecture and reproductive structure. This coupling of cellular ploidy dynamics with agronomic performance suggests that SHGD is not merely a reproductive rescue mechanism but a broader developmental regulator affecting plant growth.

Importantly, the high level of HMF in A427, to which major loci such as *qshgd1* have been shown to contribute (Molenaar et al., 2019; Trampe et al., 2020), may not be explained solely by meiotic restitution. Our observations are consistent with developmentally increasing somatic diploidization during vegetative growth, generating chimeric tissues in which diploidized cell fractions become progressively enriched. The mechanism underlying this enrichment remains unresolved. Possible explanations include greater proliferative competence of diploidized cells, developmental selection among competing cell lineages within meristematic tissues, and genome-dosage-dependent effects on cell and organ growth. The present flow-cytometric data cannot distinguish among these alternatives. Therefore, the increase in diploid cell fractions should be interpreted as evidence of developmental enrichment rather than direct proof of cell competition, selective amplification, or a specific meristematic mechanism. While causality cannot be formally established, the temporal ordering of somatic divergence prior to tassel development supports a developmental contribution of somatic ploidy changes to HMF.

These findings do not exclude additional mechanisms. Even within meiotic or mitotic categories, multiple unrelated molecular routes may lead to functional fertility restoration. SHGD should therefore be conceptualized not as a single genetic pathway but as a spectrum of mechanistic solutions that converge on restored diploid gene dosage in reproductive lineages. This multi-pathway model helps explain incomplete penetrance within genetically uniform haploid populations and the continuum from sterility to full fertility observed in our dataset.

From a breeding perspective, distinguishing between meiotic and mitotic routes to HMF has practical implications. Chemical genome doubling remains a major bottleneck in DH production systems (Chaikam et al., 2019). In the present A427 dataset, stomatal length at the fifth leaf stage showed strong apparent discrimination between plants subsequently classified as fertile and sterile, suggesting that early vegetative morphology may capture developmental differences associated with somatic ploidy transitions. Because this analysis was retrospective and was not independently validated, stomatal length should currently be considered a promising candidate indicator rather than an established predictor for routine breeding. Validation across independent populations, genetic backgrounds, and environments is required before a general selection threshold can be recommended.

Together, our results support a model in which SHGD is associated with developmentally structured and mechanistically heterogeneous changes in nuclear DNA content. In the high-HMF genotype A427, the observed vegetative enrichment of 2C and 4C nuclear fractions is consistent with a contribution of somatic/mitotic genome doubling, while meiotic restitution may operate in parallel and cannot be excluded by the present data. Several limitations should be considered. First, the developmental and discriminatory analyses were centered on A427, and the magnitude and timing of these associations should not be assumed to apply across diverse maize germplasm. Second, direct cytological examination of pollen mother-cell meiosis was not performed, preventing quantitative separation of somatic/mitotic doubling from possible meiotic restitution. Third, although the experiments were independently repeated over three years, plants were evaluated under controlled greenhouse conditions; genotype-by-environment effects under field conditions remain to be tested. Finally, the ROC analyses were developed and evaluated using the same dataset and therefore require validation in independent populations. These limitations define important priorities for future studies aimed at testing whether endogenous genome doubling and associated early phenotypes can be reliably exploited without chemical intervention.

This reinterpretation reframes SHGD as a lineage-dependent developmental buffering process that mitigates haploid frailty and provides a conceptual framework for breeding strategies that exploit endogenous genome doubling without chemical intervention. More broadly, these findings position SHGD within emerging frameworks of genome dosage compensation and developmental robustness in plant systems.

## Data Availability

The original contributions presented in the study are included in the article/**Supplementary Material**. Further inquiries can be directed to the corresponding author.

## Supplementary material

Supplementary Figure 1 Flow cytometric profiles of L1 leaf nuclei from A427 (n) plants (Fertile).

Supplementary Figure 2 Flow cytometric profiles of L5 leaf nuclei from A427 (n) plants (Fertile).

Supplementary Figure 3 Flow cytometric profiles of L10 leaf nuclei from A427 (n) plants (Fertile).

Supplementary Figure 4 Flow cytometric profiles of L1 leaf nuclei from A427 (n) plants (Sterile).

Supplementary Figure 5 Flow cytometric profiles of L5 leaf nuclei from A427 (n) plants (Sterile).

Supplementary Figure 6 Flow cytometric profiles of L10 leaf nuclei from A427 (n) plants (Sterile).

Supplementary Figure 7 Flow cytometric profiles of L15 leaf nuclei from Wf9 (n) plants (Sterile).

Supplementary Figure 8 Flow cytometric profiles of L15 leaf nuclei from W22 (2n) plants (Fertile).

Supplementary Figure 9 SL in diploid W22 plant, mean SL = 163.79 µm.

Supplementary Figure 10 SL in haploid Wf9 plant, SL mean = 79.67 µ.

Supplementary Figure 11 A427 (n) male fertile.

Supplementary Figure 12 A427 (n) male sterile.
