# Supplementary material for: Somatic genome doubling determines fertility in maize haploids of inbred A427
Source: Front Plant Sci. 2026 Sep 21;17:1937834. doi: 10.3389/fpls.2026.1937834 (PMC13635341; doi:10.3389/fpls.2026.1937834)
Supplement: Supplementary file 1 [file Supplementaryfile1.docx]

**Supplementals Figures and Table**

Supp. Fig. 1. Flow cytometric profiles of L1 leaf nuclei from A427 (n) plants (Fertile)

Supp. Fig. 2. Flow cytometric profiles of L5 leaf nuclei from A427 (n) plants (Fertile).

Supp. Fig. 3. Flow cytometric profiles of L10 leaf nuclei from A427 (n) plants (Fertile).

Supp. Fig. 4. Flow cytometric profiles of L1 leaf nuclei from A427 (n) plants (Sterile).

Supp. Fig. 5. Flow cytometric profiles of L5 leaf nuclei from A427 (n) plants (Sterile).

Supp. Fig. 6. Flow cytometric profiles of L10 leaf nuclei from A427 (n) plants (Sterile).

Supp. Fig. 7. Flow cytometric profiles of L15 leaf nuclei from Wf9 (n) plants (Sterile).

Supp. Fig. 8. Flow cytometric profiles of L15 leaf nuclei from W22 (2n) plants (Fertile)

Supp. Fig. 9. SL in diploid W22 plant, mean SL = 163.79 µm

Supp. Fig. 10. SL in haploid Wf9 plant, SL mean = 79.67 µ

Supp. Fig. 11. A427 (n) male fertile	Supp. Fig. 12. A427 (n) male sterile
